# Epidemiology of forest malaria in central Vietnam: a large scale cross-sectional survey

**DOI:** 10.1186/1475-2875-4-58

**Published:** 2005-12-08

**Authors:** Annette Erhart, Ngo Duc Thang, Phan Van Ky, Ta Thi Tinh, Chantal Van Overmeir, Niko Speybroeck, Valerie Obsomer, Le Xuan Hung, Le Khanh Thuan, Marc Coosemans, Umberto D'alessandro

**Affiliations:** 1 Prince Leopold Institute of Tropical Medicine, Nationalestraat 155, 2000 Antwerp, Belgium; 2 National Institute for Malariology, Parasitology and Entomology, Luong The Vinh street, BC 10200 Tu Liem district, Hanoi, Vietnam; 3 Provincial Centre for Malariology, Parasitology and Entomology, 156 Ngo Gia Tu Street, Phan Rang city, Ninh Thuan province, Vietnam

## Abstract

In Vietnam, a large proportion of all malaria cases and deaths occurs in the central mountainous and forested part of the country. Indeed, forest malaria, despite intensive control activities, is still a major problem which raises several questions about its dynamics.

A large-scale malaria morbidity survey to measure malaria endemicity and identify important risk factors was carried out in 43 villages situated in a forested area of Ninh Thuan province, south central Vietnam. Four thousand three hundred and six randomly selected individuals, aged 10–60 years, participated in the survey. Rag Lays (86%), traditionally living in the forest and practising "slash and burn" cultivation represented the most common ethnic group. The overall parasite rate was 13.3% (range [0–42.3] while *Plasmodium falciparum *seroprevalence was 25.5% (range [2.1–75.6]). Mapping of these two variables showed a patchy distribution, suggesting that risk factors other than remoteness and forest proximity modulated the human-vector interactions. This was confirmed by the results of the multivariate-adjusted analysis, showing that forest work was a significant risk factor for malaria infection, further increased by staying in the forest overnight (OR= 2.86; 95%CI [1.62; 5.07]). Rag Lays had a higher risk of malaria infection, which inversely related to education level and socio-economic status. Women were less at risk than men (OR = 0.71; 95%CI [0.59; 0.86]), a possible consequence of different behaviour. This study confirms that malaria endemicity is still relatively high in this area and that the dynamics of transmission is constantly modulated by the behaviour of both humans and vectors. A well-targeted intervention reducing the "vector/forest worker" interaction, based on long-lasting insecticidal material, could be appropriate in this environment.

## Introduction

Controlling malaria in forested areas remains a challenge in many parts of Asia and South America [1-7]. In Vietnam, forest malaria occurs in 16 provinces (out of 64) situated in the central part of the country (11 in the Central area, 4 in the western highlands and 1 in the south-eastern region). According to the figures reported by the National Malaria Control Program (NMCP), about half of the total malaria cases, more than 90% of the severe cases and almost 95% of malaria deaths occur in these 16 forested provinces [8,9]. In a previous community-based study [5], regular forest activity was a strong risk-factor for malaria infection and its population-attributable fraction was estimated at 53%. Workers, when staying in the forest overnight, do not usually sleep under insecticide-treated bed nets (ITN) and are therefore exposed to infection. Moreover, due to the behaviour of the main vector *Anopheles dirus *(early biting, exophagy and exophily), neither ITNs nor indoor spraying seem to be suitable control measures [10]. New interventions targeted to forest workers are urgently needed and should be tested in field trials [11-13].

A cluster randomized trial to test the protective efficacy of Long Lasting Insecticidal Hammocks (LLIH) in controlling forest malaria was launched in 2004 in collaboration with the NMCP and it is still ongoing. According to the expected impact of the intervention, estimated on the basis of previously collected epidemiology data [5], 20 clusters of about 1,000 inhabitants each were identified in Ninh Thuan province, one of the poorest and more endemic provinces, on the basis of a preliminary screening survey carried out in 43 villages. This large scale-survey allowed the analysis of the spatial and temporal distribution of the malaria infections over a large forested area and the confirmation of a previous risk-factor analysis carried out in a similar but much more limited setting [5]

## Materials and methods

### Study area and population

**Figure 1:**
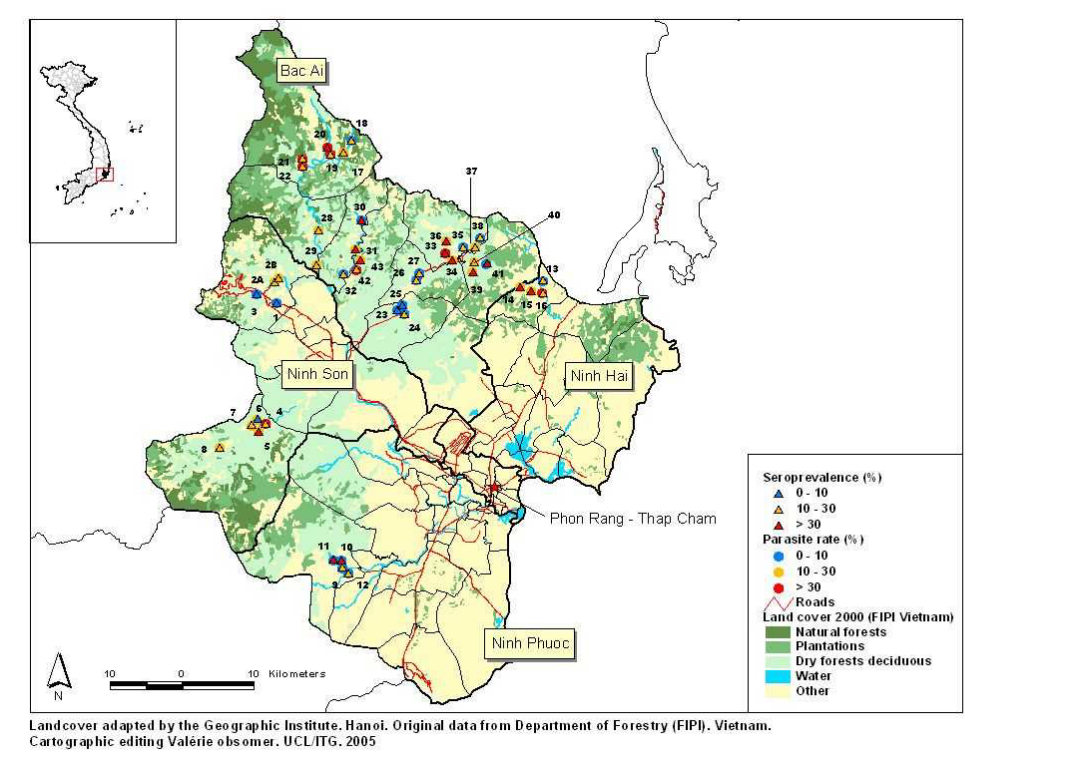
Land-cover map Ninh Thuan province with parasite rate and malaria seroprevalence for the 43 study villages-Nov.2003 (see details, Table 1).

**Figure 2:**
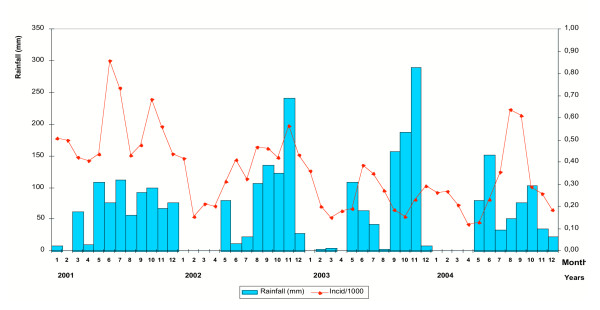
Evolution of rainfall and monthly malaria incidence in Ninh Thuan province: 2001–2004 (Data from Center of Malariology, Parasitology and Entomology, Ninh Thuan province).

The survey was carried out from November to December 2003 in Ninh Thuan province, located on the southern coast of Central Vietnam (Figure 1). Forty three villages corresponding to the forested and mountainous part (north-west) of the province and with the highest annual malaria morbidity and mortality figures according to the Provincial Malaria Station were selected. The population is distributed over 12 communes located in 4 districts (Bac Ai, Ninh Son, Ninh Phuoc and Ninh Hai) and is mainly inhabited by the Rag Lays, traditionally nomadic but recently settled in permanent villages. Most people are farmers, cultivating maize in forest fields or and rice around the villages, and collecting forest products. The dry season lasts from December to April, the rainy season from May to November with one of the lowest mean annual rainfalls in the country (<800 mm/y). The monthly mean temperature is 25–30°C, with an annual mean of 27.3°C. The mean relative humidity over the past 3 years has been 74.7%, ranging from 70 to 80% throughout the year. Malaria transmission is perennial with 2 peaks (May-June and October-November), the monthly incidence of malaria cases following closely the monthly rainfalls (Figure 2). The two main malaria vector species are *An. dirus A *and *Anopheles minimus A *[14].

### Data collection

Households were numbered and a full census carried out. A sample size of 150 individuals per cluster of 1,000 inhabitants was calculated at 5% significance level assuming a 5% parasite rate with 3.5% precision. Households were randomly chosen and within each of them, an individual aged 10 to 60 years was randomly selected (the latter age range was estimated to represent the active population most at risk of malaria [5]). Socio-demographic data, risk factors for malaria infection as well as current prevention methods were collected on pre-coded standardized questionnaires. At clinical examination, axillary temperature and spleen size were measured. Blood samples for parasitaemia (thick film) and *P. falciparum *antibodies (filter paper) were collected. Suspected malaria cases were treated with a 7-day course of artesunate according to the national guidelines; other common diseases were treated accordingly.

Village coordinates were recorded using a Geographic Position System or GPS (eTrex Summit, GARMIN Corporation) and later reported on the land-cover digital map for the year 2000 provided by the Institute of Geography – Hanoi (adapted from land-cover map compiled by the Department of Forestry – FIPI). The original map with 22 land-cover classes was simplified into the 5 following categories:

***Natural forests ***– including rich forest, mixed forest, medium forest, wood and bamboo mixed forests, bamboo forest and clear forest;

***Plantations ***– including different types of plantations;

***Dry forest deciduous***;

***Other ***– including rice fields, land tenure, habitat with garden, swamps or wetlands, and different types of bare land;

***Water ***– including natural river & lake.

Elevation varied from 10 m (Phuoc Chien commune) to 323 m above the sea-level in Phuoc Binh.

The numbers presented on the map refer to the village unique identifier. Parasite rates and seroprevalences for each village (Table 1) were reported onto the map with different coloured symbols corresponding to the following categories: 0–10%; 10–30%; = 30% (Figure 1).

**Table 1:** Parasite rate and malaria seroprevalence of the 43 study villages (see map Figure 1)

**Village Code**	**Village**	**Commune**	**District**	**Parasite rate (%)**	**Sero-prevalence (%)**
1	Lap La	Lam Son	Ninh Son	3,7	6,1
2A	Tam Ngan1	Lam Son	Ninh Son	10,4	15,4
2B	Tam Ngan2	Lam Son	Ninh Son	10,4	15,4
3	Thon Gon	Lam Son	Ninh Son	1,2	6,9
4	Thon Do	Ma Noi	Ninh Son	30,8	21,2
5	Ha Zai	Ma Noi	Ninh Son	24,7	49,4
6	Thon U	Manoi	Ninh Son	13	7,7
7	Ya Rot	Ma Noi	Ninh Son	15,4	10,3
8	Ta Noi	Ma Noi	Ninh Son	26,7	22,7
9	Ro On	Phuoc Ha	Ninh Phuoc	1,5	15,2
10	Gia	Phuoc Ha	Ninh Phuoc	8,7	38,5
11	La A	Phuoc Ha	Ninh Phuoc	5,5	42,7
12	Tra No	Phuoc Ha	Ninh Phuoc	6,2	17,1
13	Ma Trai	Phuoc Chien	Ninh Hai	3,2	23,2
14	Tap La	Phuoc Chien	Ninh Hai	14,8	58,3
15	Dau Suoi A-B	Phuoc Chien	Ninh Hai	21,1	36,1
16	Dong Thong	Phuoc Chien	Ninh Hai	32,1	20,2
17	Bac Ray 2	Phuoc Binh	Bac Ai	22,7	18,7
18	Bac Ray 1	Phuoc Binh	Bac Ai	9,4	26,9
19	Bo Lang	Phuoc Binh	Bac Ai	41,7	26,8
20	Gia E	Phuoc Binh	Bac Ai	34,1	31,8
21	Hanh Rac 2	Phuoc Binh	Bac Ai	42,3	25
22	Hanh Rac 1	Phuoc Binh	Bac Ai	39,5	21,9
23	Suoi Ro	Phuoc Chinh	Bac Ai	9,7	4,8
24	Suoi Kho	Phuoc Chinh	Bac Ai	5,9	20,8
25	Nui Ray	Phuoc Chinh	Bac Ai	0	2,1
26	Ta Lu (1,2,3)	Phuoc Dai	Bac Ai	2,8	10,3
27	Ma Hoa	Phuoc Dai	Bac Ai	6,9	25
28	Ta Lot	Phuoc Hoa	Bac Ai	23,3	29,3
29	Cha Panh	Phuoc Hoa	Bac Ai	12,2	13,1
30	Ma Lam	Phuoc Tan	Bac Ai	6,7	75,6
31	Ma Ty	Phuoc Tan	Bac Ai	23,6	37,3
32	Da Trang	Phuoc Tan	Bac Ai	4,4	22,5
33	Cha Dung1	Phuoc Thang	Bac Ai	36,6	62,4
34	Ma Ty	Phuoc Thang	Bac Ai	11,8	49
35	Ha La Ha	Phuoc Thang	Bac Ai	5,3	12,3
36	Ma Oai	Phuoc Thang	Bac Ai	15,1	36,2
37	Ma Ro	Phuoc Thanh	Bac Ai	16,7	27,8
38	Da Ba Cai	Phuoc Thanh	Bac Ai	6,7	23,3
39	Suoi Lo	Phuoc Thanh	Bac Ai	14	38
40	Manai	Phuoc Thanh	Bac Ai	26,7	21,3
41	Mazu	Phuoc Thanh	Bac Ai	9,6	35,2
42	Da Ban	Phuoc Tien	Bac Ai	35	19
43	Suoi Rua	Phuoc Tien	Bac Ai	14,3	59,2

### Laboratory methods

Thick films were stained with a 5% Giemsa solution for 30 minutes. Parasite densities were computed based on the number of asexual forms and gametocytes per 200 white blood cells (WBCs), assuming a mean WBC count of 8,000/μl. A slide was classified as negative if no Plasmodium asexual form was found after counting 1,000 WBCs. Filter papers (Whatman N°3, Kent, United Kingdom) were stored at -20°C, and Indirect Fluorescent Antibody Tests (IFAT) were carried out to determine the total immunoglobulin titres against *P. falciparum *as described previously [15]. *P. falciparum *antigen was prepared from *in vitro *cultures of an isolate originating from a patient in Southern Vietnam. Negative control serum was obtained by pooling the sera of five malaria-free individuals; positive control serum, by pooling the sera of 5 patients who had already suffered several malaria episodes. The serum dilutions ranged from 1:80 to 1:640. Both slide and IFAT reading were blinded and carried out at NIMPE (National Institute of Malariology, Parasitology and Entomology), Hanoi. Quality control was performed at ITM (Institute of Tropical Medicine), Antwerp, on all positive and 10% negative samples and all discrepant results were re-read and confirmed by a third technician.

### Case definition

- Suspected malaria: patient with typical malaria symptoms without microscopic diagnosis;

- Malaria infection: positive slide with Plasmodium asexual forms, regardless of symptoms;

- Clinical malaria: fever (BT≥37.5°C) and/or history of fever in the past 48 hours with a positive blood slide;

- Positive IFAT: titre ≥ 1/80. The immune response to an active malaria infection is species and strain specific, and lasts 6–9 months without additional exposure [16,17]. Usually titres above 1:20 are considered positive. However, we chose a more specific screening titre of 1:80.

- Current & recent malaria infections: to perform the multivariate adjusted risk-factor analysis for malaria infections (Table 4) all cases with either a positive slide and/or a positive IFAT were combined.

**Table 4:** Risk factor analysis for malaria infections (positive slide and/or positive IFAT (1/80)): uni- and multi-variate adjusted analysis using survey logistic regression (n = 1,434)

Risk factors	Prevalence (%)	Cases (n/N)	Unadjusted		Multivariate Adjusted	
				
			OR	95%CI	OR	95%CI
				
**Total**	33.3	1,434/4,306				
**Sex:**						
- Male	35.8	659/1841	1		1	
- Female	34.1	775/2465	0.82	[0.71; 0.96]°	0.71	[0.59; 0.86]°
**Age (y):**						
- 10–20	30.6	293/959	1			
- 20–30	33.5	501/1494	1.15	[0.95; 1.39]		
- 30–40	35.6	346/972	1.26	[1.03; 1.53]°		
- 40–50	36.0	194/539	1.28	[1.09; 1.56]°	-	-
- 50-max	29.2	100/342	0.94	[0.67; 1.32]		
**Ethnic group:**						
- Kinh	13.2	30/227	0.26	[0.17; 0.43]°°	0.67	[0.29; 1.52]
- Rag Lay	36.6	1355/3705	1		1	
- Others	13.1	49/374	0.26	[0.12; 0.58]°	0.38	[0.22; 0.66]°
**Education:**						
- No	40.6	798/1968	1		1	
- Primary	30.1	556/1849	0.63	[0.50; 0.80]°°	0.68	[0.52; 0.88]°
- Secondary +	16.4	80/489	0.29	[0.22; 0.38]°°	0.46	[0.34; 0.64]°°
**Bed-net availability:**						
- No net	44.6	41/92	1.83	[1.05; 3.21]°		
- Untreated nets	40.9	9/22	1.58	[0.70; 3.54]	-	
- > 2pers./net	34.3	948/2761	1.19	[0.98; 1.45]		
- 1–2 pers./net	30.5	436/1431	1			
**House structure:**						
- Bamboo	40.5	883/2183	1		1	
- Wooden	27.6	338/1224	0.56	[0.42; 0.76]°°	0.85	[0.68; 1.08]
- Dried mud	29.7	70/236	0.62	[0.40; 0.97]°	0.62	[0.41; 0.94]°
- Bricks	21.6	143/663	0.40	[0.30; 0.56]°°	0.81	[0.62; 1.07]
**Socio-economic status:**						
- No radio, TV, moto	37.8	1037/2742	1		1	
- Only radio	34.6	155/448	0.87	[0.65; 1.17]	0.97	[0.74; 1.27]
- TV only	27.6	82/297	0.63	[0.38; 1.03]°	0.73	[0.48; 1.09]
- TV + radio	20.7	34/164	0.43	[0.26; 0.71]°	0.60	[0.39; 0.93]°
- Moto (+/-radio +/-TV)	19.2	126/655	0.39	[0.28; 0.55]°°	0.54	[0.37; 0.78]°
**Profession:**						
- Other work	14.7	65/441	1			
- Forest work	36.5	1245/3412	3.32	[1.39; 3.42]°°	-	
- No (children, students, old)	27.4	124/453	2.18	[2.36; 4.68]°		
**Forest work:**						
- Other work	14.7	65/441	1		1	
- Occasional (+child/retired)	25.9	258/993	2.03	[1.14; 3.18]°	1.48	[1.01; 2.17]°
- Regular, no sleep	35.7	681/1906	3.22	[2.09; 4.94]°°	1.76	[1.05; 2.94]°
- Regular + sleep	48.8	327/670	5.51	[3.42; 8.90] °°	2.86	[1.62; 5.07]°
- Missing data	34.8	103/296	-			

°p < 0.05

°°p < 0.001

### Statistical analysis

Data were entered in Epi Info 6.04 (CDC, Atlanta; WHO, Geneva 1996) and analysed in STATA 8 software (Stata Corp.2003, College Station, TX). Malaria parasite rate and sero-prevalence were computed by village and a survey chi-square test ("svytab" command in STATA) was used to test for significant differences for these two variables between villages of each commune and between the communes themselves. A survey logistic regression ("svylogit" command in STATA) was carried out taking into account the survey characteristics, with villages as primary sampling units and communes as strata. Uni- and multivariate adjusted analysis for the risk of malaria infection during the rainy season was carried out. Therefore, current (positive slide) and recent (IFAT ≥ 1:80) malaria infections were combined in order to include most malaria infections during the previous six months up to the time of the survey. The primary exposure was forest work and this variable was coded as follows: 1) not working in forest (other job); 2) occasionally (less than weekly); 3) regularly (at least weekly), but not staying overnight; 4) regularly working in forest and sleeping there at various frequency. The category – no profession, i.e. children, students and retired people, was included in the category occasionally working in forest since they do so with their family.

The Ethical Committee of ITM, Antwerp and that of NIMPE, Hanoi approved the study (Ethical clearance registration n°: OG 018). Informed consent was received from all village leaders and People Committees after explaining the study objectives and methodology. Selected individuals were informed and invited to be part of the survey some days in advance but were free to refuse. Other people not chosen but being sick at the time of the survey were examined and treated accordingly but were not included in the survey database.

## Results

### The study population (Table 2)

**Table 2:** Baseline characteristics of the study population and forest workers

**Study population, n = 4,306**	N (%)	95%CI
**Sex:**		
- Male	1,841 (42.8)	[40.7; 44.8]
- Female	2,465 (57.2)	[55.2; 59.3]
**Age categories:**		
- 10–19 y	959 (22.3)	[21.0; 23.6]
- 20–29 y	1,494 (34.7)	[33.4; 36.0]
- 30–39 y	972 (22.6)	[20.9; 24.3]
- 40–49 y	539 (12.5)	[11.4; 13.7]
- >50 y	342 (7.9)	[6.5; 9.4]
**Ethnic groups:**		
- Rag Lay	3,705 (86.0)	[83.1; 89.0]
- Kinh	227 (5.3)	[3.0; 7.5]
- Ko'ho	349 (8.1)	[3.9; 12.3]
- Others (Cham, Ede, Chu,)	25 (0.6)	[0.3; 0.9]
**Education level:**		
- None	1,968 (45.7)	[42.4; 49.0]
- Primary school	1,849 (42.9)	[40.0; 45.9]
- Secondary school or university	489 (11.6)	[9.4; 13.3]
**Bed-net coverage, **median [range]	2.5 pers/net	[0.2; 11]
**Bed-net categories:**		
- No net	92 (2.1)	[1.1; 3.2]
- Untreated nets	22 (0.5)	[0; 1.1]
- >2 pers./net (treated)	2,761(64.1)	[60.3; 68.0]
- 1–2 pers./net ( " )	1,431(33.2)	[29.4; 37.1]
**House structure:**		
- Thatched bamboo	2,183 (50.7)	[45.3; 56.1]
- Wooden boards	1,224 (28.4)	[23.4; 33.4]
- Dried mud	236 (5.5)	[2.9; 8.0]
- Bricks	663 (15.4)	[12.7; 18.1]
**Socio economic level:**		
- No radio, no TV, no motorbike	2,742 (63.7)	[58.8; 68.5]
- Only radio (no TV, no moto)	448 (10.4)	[8.7; 12.1]
- TV (+/-radio)	461 (10.7)	[8.3; 13.1]
- Motorbike (+/- radio or TV)	655 (15.2)	[12.0; 18.4]
**Profession:**		
- Forest work (farming & other)	3,412 (79.2)	[76.1; 82.3]
- Other (rice farmer, teacher, health staff...)	441 (10.2)	[7.4; 13.1]
- None (children, students, retired people)	453 (10.5)	[9.1; 12.0]

***Forest workers, n = 3,412***		
***Type of forest activity:***		
- *Farming*	*3,395 (99.5)*	*[99.2; 99.8]*
- *Exploitation forest products (fishing, hunting, etc...)*	*15 (0.4)*	*[0.1; 0.7]*
- *Other (cow breeding, guardian)*	*2 (0.06)*	*[0; 0.1]*
***Median number of days/month spent in forest:***	*27*	*range [0;30]*
***Sleeping in the forest:***		
- *Yes*	*1,003 (29.4)*	*[24.0; 34.8]*
- *No*	*2409 (70.6)*	*[65.2; 76.0]*
***Median number of nights/month spent in forest****(n = 1003):*	*12*	*range [1; 30]*
***Sleeping place in the forest:***		
- *Outside*	*112 (11.2)*	*[5.1; 17.2]*
- *Plot huts*	*891 (88.8)*	*[82.8; 94.9]*
***Using hammocks when sleeping in forest:***		
- *Yes (hammock nets = 27)*	*85 (8.5)*	*[4.8; 12.2]*
- *No*	*918 (91.5)*	*[87.8; 95.2]*
***Using preventive an/or standby treatment when going to forest:***		
- *Yes*	*80 (2.3)*	*[0.8; 3.9]*
- *No*	*3332 (97.7)*	*[96.1; 99.2]*

4,306 (92%) of the 4,679 selected individuals participated to the survey with a sex ratio of 1.3 in favour of females. The Rag Lay ethnic group represented the vast majority of the study population (86%), and educational level was generally low since almost half of the population had never attended school. However, communication was easy since most people (91.7%) could speak Vietnamese. Bed-nets (usually double nets) were widely used (98% of households) with a median of 2.5 persons/net. Most of the nets had been treated with insecticide the previous year, but no indoor spraying had been done. The socio-economic status was very low since half of the population was living in houses made of thatched bamboo and more than 60% owned none of the three items asked for (radio, television, or motorbike). About 80% of the study population was defined as forest workers, i.e. their main source of income was from forest activities (farming, hunting, logging, etc...).

### Forest work characteristics (Table 2)

Farming represented the main forest activity (99.5%), mostly maize and manioc, rice being generally cultivated around the villages. During the month prior to the survey, forest workers spent a median of 27 days in the forest. Most of them usually went to the forest with other family members, including children and elderly people (categorized in our study as "no profession"). About 30% of the forest workers stayed sometimes overnight in the forest (or forest fields), with a median of 12 nights during the month prior to survey. Important seasonal variations exist: April (preparation of fields before rains), July-August (first harvest) and for some villages November (second harvest) are the periods of highest forest activity. Few forest workers reported using hammocks (because they could not afford them) and hammock-nets were even rarer. Other preventive measures, such as standby and/or preventive malaria treatment, were rarely used (2.3%).

### Malariometric indices (Table 3)

**Table 3:** Malariometric indices

**Indicators (N = 4,306)**	**n (%)**	**[95%CI]**
- Prevalence of fever cases	668 (15.5)	[12.5; 18.5]
- Fever cases attributable to malaria	125 (18.7)	[13.7; 23.7]
- Spleen rate (n = 301)	301 (7.0)	[4.3; 9.7]
- *Median age if enlarged spleen [range]*	*30 y*	*[11; 60]*
- Mean parasite rate	571 (13.3)	[10.6; 15.9]
- Species distribution (n = 571):		
*P. falciparum*	*276 (48.3)*	*[41.3; 55.3]*
*P. vivax*	*261 (45.7)*	*[39.0; 52.4]*
*P. malariae*	*3 (0.5)*	*[0; 1.1]*
*Mixed infections*	*31 (5.4)*	*[3.2; 7.6]*
- Parasite density/μl (geometric mean):		
*P. falciparum*	178.3	[141.2; 225.2]
*P. vivax*	51.5	[44.0; 60.3]
- Proportion of asymptomatic infections	423 (74.1)	[67.8; 80.4]
- *P. falciparum *sero-prevalence (N = 4,253)	1,085 (25.5)	[20.7; 30.3]

Six hundred sixty eight (15.5%) individuals had fever at the time of the survey, 125 (18.7%) of them being attributable to malaria. The spleen rate was 7% (all but two individuals had a Hackett Class 1) and the median age among people with enlarged spleen was 30 years (range [11–60]). The mean parasite rate (PR) was 13.3%, ranging from 0 to 42.3% across the 43 villages, with a large majority of asymptomatic infections (74.1%). *P. falciparum *and *Plasmodium vivax *were equally represented, though the mean parasite density was significantly lower for the latter, and only three patients were found positive with *Plasmodium malariae*. About 5% (31) of the infections were mixed, mostly *P. falciparum *with *P.vivax *(28), one patient had the three species, two had *Pf+Pm *and one *P.v +P.m*.

*P. falciparum *seroprevalence (SPV) was generally high, 25.5%, ranging from 2.1 up to 75.6% across villages.

### Mapping of PR and SPV (Figure 1 & Table 1)

All 43 villages were located in places classified as 'dry forests deciduous' or 'others', situated at various distance from the natural forest. Within each commune the respective distances to the different types of vegetations are similar between the villages, since the latter are usually close to each other, i.e.12 km (except in Phuoc Binh commune where the 6 villages stretch over almost 10 km and in Phuoc Tan, over 5 km).

The mapping of PR and SPV shows an extremely patchy distribution without geographic clustering or trends per commune, per direction or elevation. Significant differences for both PR and SPV were found between the 12 communes, and between villages in almost all (10/12) communes (F (design based) p < 0.05)).

Four villages (n°1-3-23-25) had both low PR and SPV, below 10%. These villages are situated in the plain, near the main district road leading to Dalat or to the district town in Phuoc Dai commune. Nui Ray was the only village among the 43 with a PR at zero. At the other extreme, four villages had a SPV above 50% (n°14-30-33-43): all of them were remote with difficult access and near to the forest. In these villages, the PR was relatively low (ranging from 6.7% in Ma Lam to 36.6% in Cha Dung), reflecting higher transmission rates during the past six months, at the time of higher forest activity. Similarly, in Phuoc Ha and in Phuoc Dai communes (villages n° 9–12 and 26, 27), all six villages had a PR<10% but a SPV>10% (from 15.2 to 42.7%). However, remoteness was not the only determining factor for high PR and SPV since some remote villages such as Tanoi (n°8) in Manoi commune and Bac Ray1 (n°18) in Phuoc Binh commune, did not have higher PR or SPV than the other villages within the same commune. Overall, more than half of the villages (24) had either a PR>10% and/or a sero-prevalence>25%. Usually the SPV was higher than the PR, though some villages had a SPV lower than the PR. This could be explained either by the higher current exposure or by the higher prevalence of *P. vivax *(as in Bo Lang or Da Ban (data not shown)).

### Risk factors for malaria infection (Table 4)

All current and recent malaria infections were combined to perform a risk-factor analysis. A total of 1,434 infections (33.3%) were included. Among risk factors identified by uni-variate analysis, Rag Lay ethnicity, education, socio-economic status and regular forest work had the strongest effect on the odds of malaria infection. After adjusting for the effect of potential confounders, regularly working and sleeping in the forest was a strong risk-factor with the odds almost three times higher than that of people not working in the forest. Even occasional forest work was a significant risk-factor for malaria (Adjusted OR = 1.48; 95%CI [1.01; 2.17]). The malaria risk was significantly lower in people living in houses made of dried mud, while wooden or brick houses were no more protective, after adjusting for socio-economic status. There was a decreasing trend for the odds of malaria infection with increasing socio-economic status, the highest category had about 50% lower malaria risk compared to the poorest one.

## Discussion

This large-scale cross-sectional survey confirms that Ninh Thuan province remains one of the highest endemic malaria provinces in Vietnam [8,9], especially in its north-western forested and hilly area where the study was carried out. The mean PR and SPV were high, respectively 13.3 and 25.5%, with upper limits at 42.3% and 75.1% respectively. Forest malaria is extensively described in different Asian [2,4,6,18] and South American countries [3,7,19], where it remains a real challenge for control programs [1]. A previous study carried out in a village located in a forested area of the neighbouring province Binh Thuan, also reported high malaria incidence (11/100 person-years) and SPV (20–25%)[5]. However, unlike the previous study, the SPV was high also in people not working in the forest, indicating that village transmission might be important. Indeed, many surveyed villages were surrounded by the forest or not far from it so that the man/vector contact could be high for all their inhabitants.

The mapping of the PR and SPV by village showed great heterogeneity between villages located in a comparable ecological setting. This suggests that factors other than environmental ones intervene in modulating the human/vector interaction. The population in this study was mainly Rag Lays, an ethnic minority that used to live and work in the forest practising "slash and burn" cultivation, previously reported as a risk-factor for malaria [6]. Moreover, Rag Lays used to be nomads in forested mountains. Although attempts to settle them down in permanent villages are ongoing, the forest where they use to collect various products (bamboo, nuts, berries, game animals and birds) remains their natural environment. This way of life exposes them to a higher risk of malaria infection than other ethnic groups.

Even after adjusting for the effect of forest work, ethnic group, age, and education, women were still significantly less at risk of malaria, confirming the results of a previous study [5]. Compared to men, women usually remain well-covered, particularly when working outside, thus reducing the risk of exposure to mosquito bites. Women go to sleep earlier (with the children under a net, if available) while men like to sit outside around a fire. Considering that *An. dirus *bites early and is a highly anthropophylic and exophagic vector, men are obviously more exposed than women.

Education was an important protective factor and there was a decreasing trend of malaria risk with increasing level of education, even after adjusting for socio-economic status and forest work. This confirms its importance in preventing diseases in general and malaria in particular. Hence, the Vietnamese national programme for poverty alleviation [13,20] which, among other strategies, provides full subsidies to ethnic minorities for their children education (primary and secondary school), might significantly contribute to reduce not only poverty but also diseases like malaria. Independently of the socio-economic status, education is a crucial factor for adherence to malaria prevention measures, and even more crucial is the education of mothers to give prompt and effective malaria treatment to small children [21-23].

Numerous studies from SEA and Africa have extensively reported poor housing conditions as a significant risk factor for malaria infections due to greater human-vector contacts, and this is especially true for endophagic vectors like *Anopheles gambiae *and *Anopheles culicifacies *[24-27]. In this study, even if the main vector is exophagic and exophilic, the house structure might still have an impact on man-vector contact since bamboo houses are usually on stilts with wide openings in the floor as well as in the walls between bamboo canes, decreasing the outdoor/indoor difference and allowing *An dirus *to bite indoors [10]. Even if wooden and bricks houses showed a trend for a protective effect, only dried mud houses had a significant and strong protective effect and this might be due to their architecture as windows are extremely small and might reduce the human/vector contact.

One advantage of this study is the dissociation of housing effect from the socio-economic status, an important point in the setting, where even rich people still prefer to live in the Rag Lay traditional stilt bamboo houses. Even though the socio-economic classification used in this study was not the result of an exhaustive in-depth analysis of family resources and levels of income, it gives nevertheless a rough idea of their purchase power, independently of the housing conditions. Thus, even if residual confounding might have occurred, it is unlikely that it would greatly change the results, since there is a clear trend for decreasing odds of malaria infections with increasing level of socio-economic status. Malaria is and remains a poverty-related disease [28-30].

The number of current malaria infections identified during the survey (571) contrasts sharply with those collected by the provincial health information system (499 cases in the whole province during the 2 months prior to the survey). The fact that 75% of the infections detected during the survey were asymptomatic raises the question of the development of immunity in this ethnic group regularly exposed to malaria infections, and/or under-reporting of cases in the HIS due to either self-treatment or treatment by traditional healers or private practitioners.

## Conclusion

This study confirms the high malaria morbidity in this area of Ninh Thuan province and the reality represented by the complex problem of "Forest malaria". The PR and SPV mapping with its patchy distribution suggested a highly variable dynamic of transmission in time and space, since various combinations of values for both variables could be observed between and within communes. This variability is driven by the constant interplay of human behavioural factors, as showed by the risk factor analysis, and vector behaviour.

Forest malaria is a complex phenomenon which results in an epidemiological mosaic [1] and more in-depth entomological and anthropological studies are necessary to understand the specific mechanisms of forest malaria transmission in Central Vietnam. In the framework of the LLIH trial entomological and anthropological studies are ongoing and will contribute to broaden our knowledge on how malaria is maintained and subsequently could be controlled in these settings.

## Conflict of interest statement

The authors hereby certify that no conflict of interest of any kind occurred in the framework of this study.

## Financial support

The present study was supported by the UBS-Optimus Foundation, Zurich (Switzerland), and the Belgium Cooperation, in the framework of a four-year project investigating the efficacy of Long Lasting Insecticidal Hammocks in preventing forest malaria. This project is the continuation of an over 10-year long bilateral collaboration between the National Institute for Malariology, Parasitology and Entomology, Hanoi and the Prince Leopold Institute of Tropical Medicine, in Antwerp.

## Authors' contributions

Dr. Thang (Assistant Project Coordinator in NIMPE) and Dr. Ky (Director of the Malaria Center in Ninh Thuan province) organized the survey, coordinated and supervised the field work (3 study teams worked simultaneously for 1 month) and the data entry. Valérie Obsomer (agronomist expert on GIS) elaborated the Ninh Thuan map, and Niko Speybroeck, (agronomist and statistician) contributed to the data analysis. Dr. Hung (Vietnamese Project Co-ordinator) helped in the study design and choice of study villages to be included in the LLIH trial, and reviewed the manuscript. Pr. Thuan (Director of NIMPE) contributed to the choice of the study sites, and contributed to review the manuscript. Pr. U.D'Alessandro (Principal investigator of the LLIH trial) contributed to all steps, from the elaboration of the project up to the final review of this article. Pr. Marc Coosemans, entomologist, reviewed the manuscript. Dr. Ta Thi Tinh and Mrs Chantal Van Overmeir processed all the filter papers with the IFAT and performed the quality controls of all blood samples results. Dr A. Erhart (Assistant Project Co-ordinator in ITM) contributed to the study design, implementation, supervision, the data analysis and wrote the manuscript.
